# A possible connection between reactive oxygen species and the unfolded protein response in lens development: From insight to foresight

**DOI:** 10.3389/fcell.2022.820949

**Published:** 2022-09-21

**Authors:** Lixiong Gao, Ni Jin, Zi Ye, Tianju Ma, Yang Huang, Hongyu Li, Jinlin Du, Zhaohui Li

**Affiliations:** ^1^ Senior Department of Ophthalmology, The Third Medical Center of Chinese PLA General Hospital, Beijing, China; ^2^ Department of Endocrinology, The Second Medical Center and National Clinical Research Center for Geriatric Diseases, The Chinese PLA General Hospital, Beijing, China

**Keywords:** lens development, ROS, UPR, lentoid body, PSCs

## Abstract

The lens is a relatively special and simple organ. It has become an ideal model to study the common developmental characteristics among different organic systems. Lens development is a complex process influenced by numerous factors, including signals from the intracellular and extracellular environment. Reactive oxygen species (ROS) are a group of highly reactive and oxygen-containing molecules that can cause endoplasmic reticulum stress in lens cells. As an adaptive response to ER stress, lens cells initiate the unfolded protein response (UPR) to maintain normal protein synthesis by selectively increasing/decreasing protein synthesis and increasing the degradation of misfolded proteins. Generally, the UPR signaling pathways have been well characterized in the context of many pathological conditions. However, recent studies have also confirmed that all three UPR signaling pathways participate in a variety of developmental processes, including those of the lens. In this review, we first briefly summarize the three stages of lens development and present the basic profiles of ROS and the UPR. We then discuss the interconnections between lens development and these two mechanisms. Additionally, the potential adoption of human pluripotent stem-cell-based lentoids in lens development research is proposed to provide a novel perspective on future developmental studies.

## 1 Introduction

The lens is a unique structure in the eyeball. With the main function of adjustably refracting light, the lens must be transparent, refractive, and elastic (Hejtmancik et al., 2015). To generate this sophisticated component, an organism needs an orchestrating embryonic developmental process that turns a mass of cells, namely, the lens placode, into what we know as the crystalline lens (Bassnett and Sikic, 2017). To some extent, lens development is simple. Only two kinds of cells are involved in this process: lens fiber cells (LFCs) and their antecedents, lens epithelial cells (LECs) (Bassnett and Sikic, 2017). This simplicity allows biologists and geneticists to use the lens as a perfect model to study complicated tissue formation and organization. However, lens development is also complex. Lens cell fate determination, precise cell migration, and timely organelle degradation all require a finely tuned gene regulatory network, which has attracted scientists interested in deciphering the underlying mechanism within this “black box.” In the past two decades, tremendous progress at the cellular and molecular levels has been made in understanding how lenses are formed. These studies have generated a complex yet incomplete picture describing factors that influence lens development from different perspectives. Signals from the extracellular microenvironment are among these factors. Human lens development involves the formation and degradation of the arteria hyaloidea, causing a microenvironmental shift favorable for lens formation (Ko et al., 1985). This process refers to the level change in an important factor that participates in the vast majority of physiological processes: oxygen. In 2019, three outstanding scientists were honored with the Nobel Prize in Physiology or Medicine for their contribution to cellular oxygen sensing, highlighting the significance of discovering the physiological and pathological influence of oxygen.

Oxygen not only participates in aerobic metabolism but also serves as the major coordinator in determining the cell fate (Mohyeldin et al., 2010). As the “byproduct” of oxygen, reactive oxygen species (ROS) are canonically regarded as the driving factors of many pathological conditions (Spector, 1995; Andersen, 2004). However, in the last two decades, growing evidence has clearly confirmed the important physiological mediating roles of ROS (Weidinger and Kozlov, 2015), including the regulation of normal development processes. Generally, low levels of ROS contribute to the maintenance of the stemness of stem cells, while relatively high levels contribute to the promotion of cell differentiation (Bigarella et al., 2014). ROS are also capable of causing endoplasmic reticulum stress, which initiates the unfolded protein response (UPR) (Koumenis et al., 2002; Romero-Ramirez et al., 2004; Koritzinsky et al., 2006; Zhang et al., 2019). The proapoptotic roles of the UPR in pathological processes have been widely studied. However, as an adaptive response, the UPR plays positive roles in physiological processes. Recent studies have also found evidence of its participation in normal organ development. Since human lens development may undergo a switch in microenvironmental oxygen concentration due to the formation and degradation of the arterial hyaloidea, we speculate that ROS/UPR may also participate in the cell fate determination of lens cells. Moreover, current studies were mainly founded on animal models that are profoundly different than humans. This review briefly summarizes the intrinsic characteristics of lens development and their possible relationship with the ROS/UPR and proposes possible future solutions to better understand the influence of the ROS/UPR during human lens development.

## 2 Major steps to form the lens and the corresponding molecular mechanism

Broadly speaking, lens development spans from the formation of zygotes until postnatal growth. Here, the review mainly discusses the development in the narrow sense, which is embryonic lens morphogenesis. The process comprises three general phases: the specification of lens progenitor cells and the formation of the lens placode, the invagination of the lens placode, and the differentiation of LFCs (Cvekl and Ashery-Padan, 2014).

### 2.1 Formation of lens placode

When a neural plate is formed, the anterior part of the neural plate “border,” which lies between the neural ectoderm and the nonneural ectoderm, is subsequently specified and known as the anterior preplacodal region (aPPR) (Litsiou et al., 2005; Grocott et al., 2012; Cvekl and Ashery-Padan, 2014). The aPPR is a sensory placode that contains a mixture of progenitor cells, including lens progenitor cells (Bailey and Streit, 2005; Sato et al., 2010). Growing evidence has shown that aPPR has a unique molecular signature (Sato et al., 2010). Upon specific signaling, progenitor cells within the aPPR migrate and converge into the adenohypophyseal, olfactory, and lens placodes (Cvekl and Ashery-Padan, 2014). Interestingly, all these placodes are initially specified as lenses, and only when the restriction of lens specification emerges can the formation of adenohypophyseal and olfactory placodes be promoted (Bailey et al., 2006). The molecular signals that drive the initial phase are combinatory actions driven by different pathways, mainly FGF, BMP, and Wnt. The FGF family plays a crucial role in the induction of the first PPRs (Ahrens and Schlosser, 2005; Litsiou et al., 2005). It was proposed that FGF family members promote the expression of “preneural” genes and regulate PPR-specific transcription factors (Grocott et al., 2012; Patthey and Gunhaga, 2014). During this stage, the expression level of BMP signaling first increases and then decreases (Kwon et al., 2010), but Wnt signaling remains consistently repressed (Litsiou et al., 2005; Patthey and Gunhaga, 2014). However, all progenitor cells within the aPPR have the ability to express PAX6. In a study of mice, deletion of *P*ax6 caused the failure of lens placode formation in mice embryos, indicating the critical status of PAX6 in subsequent lens placode formation (Huang et al., 2011). Similarly, FGF and BMP signaling is indispensable for specific PPR formation into the lens placode. Conditional deletion of *F*gfr1 and *F*gfr2 in mice resulted in the formation of small lenses, while conditional deletion of both *B*mpr1a and *F*gfr2 prevented lens formation (Garcia et al., 2011). More importantly, in another study of mice, deletion of *B*mp4, a member of the BMP family, completely blocked the formation of lens placodes (Furuta and Hogan, 1998). Some other factors, including SIX3, OTX2, and PAR3, although not typically specific to this region, also participate in lens placode formation (Purcell et al., 2005; Ogino et al., 2008; Melo et al., 2017). However, during this stage, Wnt signaling can inhibit the establishment of aPPR and promote neural crest formation (Saint-Jeannet and Moody, 2014). Taken together, the specification of lens progenitor cells within the aPPR and the subsequent formation of lens placodes under specific signals and critical factors are the intrinsic properties of the initial phase of lens development.

### 2.2 Invagination of lens placodes

After the lens placode is formed, the invagination process is initiated and is synchronized with the invagination of the optic vesicle to form the optic cup. If induction of the lens cells is the principal event in the first phase, then lens cell proliferation and morphological construction are the core events in the second phase. The cells within the lens placode proliferate to increase its thickness and simultaneously accumulate extracellular matrix to confine the “area” of the lens (Cvekl and Ashery-Padan, 2014). When reaching a certain number, these columnar placodal cells elongate and transform into conical-shaped cells (Sawyer et al., 2010) and begin invaginating to form lens vesicles. This transformation process, also known as apical constriction or cell wedging, is an important process in epithelial invagination during organ development, including lens development (Chauhan et al., 2015). Studies of mice show that circumferential contraction of the actomyosin cytoskeleton in lens cells, which requires RhoA, Rho-kinase, Shroom3, and p120-catenin, ultimately decreases the apical bicellular junctional length and results in lens invagination (Plageman et al., 2010; Lang et al., 2014). Among these molecules, Shroom3 is necessary for the apical localization of F-actin and myosin Ⅱ, which are critical components for apical constriction. Additionally, the expression of Shroom3 is regulated by PAX6, the crucial transcription factor in lens induction, as mentioned earlier (Plageman et al., 2010), indicating the indispensable role of Shroom3 in the second phase of lens development. Recently, a study of mice showed that the formation of multicellular actomyosin cables is essential for the invagination of lens placodes (Houssin et al., 2020). These contractile factors converge into mechanical forces and promote the invaginating process (Hosseini and Taber, 2018). However, as invagination continues, a Rho GTPase, CDC42, antagonizes the contractile function that is promoted by Shroom3 and ensures the elongation of lens cells in a study of mice (Muccioli et al., 2016). Additionally, the core member of the Par complex, PAR3, was found to reduce the junctions of actomyosin contraction and to play an actomyosin-activation-regulation role in the lenses of mice (Houssin et al., 2020). In summary, the orchestration of these opposing processes under comprehensive cellular and molecular factors carefully pushes the invagination of the lens placode forward to form lens vesicles and guarantees its strict direction, which is the core event in the second phase of lens development.

### 2.3 Differentiation of LFCs

Characterized by both transparency and refractive power, the crystallin lens mainly consists of mature LFCs that contain a large amount of crystallin proteins and few organelles. Thus, once the invagination is complete, the lens pit closes to form the lens vesicle, which is a hollow structure containing lens progenitor cells. The anterior cells form LECs, and the LECs exit the cell cycle and undergo the initial LFC differentiation process toward the posterior part of the vesicle, which is the core event in the following developmental stage (Audette et al., 2017). RNA-sequence analysis revealed profound differences in gene expression in LECs and LFCs (Zhao Y. et al., 2018), indicating a complex transcriptional transition during LFC differentiation. Notably, there are two phenotypes but different LFC differentiation statuses. Primary LFC differentiation is undertaken by posterior embryonic lens progenitor cells, which later form the lens nucleus, and secondary LFC differentiation transpires with equatorial LECs, which later form the lens cortex (Cvekl and Zhang, 2017). Both LFC differentiation mechanisms contain three major processes: lens cell elongation (Rao and Maddala, 2006), crystallin protein production, intracellular organelle degradation (Bassnett, 2009), and cell-to-cell communication establishment (Martinez and de Iongh, 2010). A study of chicken embryos showed that microtubules play crucial roles in lens differentiation, mainly influencing elongation processes by interacting with actomyosin and N-cadherin junctions (Logan et al., 2018). (Please refer to the concise review (Audette et al., 2017).) FGF signaling also plays a very important role in LFC differentiation. It is widely accepted that a low dose of FGF mainly promotes the proliferation of LECs, but a high dose promotes LFC differentiation (Lovicu and McAvoy, 2005; Cvekl and Ashery-Padan, 2014; Audette et al., 2017; Cvekl and Zhang, 2017). Abundant studies of mice and rats have reported the specific mechanisms of FGF signaling. FGF facilitates LFC elongation through the CRK/FRS2/SHP2/GRB2 complex (Collins et al., 2018), Hippo–Yap signaling (Dawes et al., 2018), and the MAPK pathway (Lovicu and McAvoy, 2001; Upadhya et al., 2013). This effect can be antagonized by SPRY and SPREAD, negative regulators of the RTK-mediated MAPK pathway (Zhao G. et al., 2018). FGF signaling also promotes the synthesis of the crystallin protein (West-Mays et al., 2010). The proto-oncogene c-Maf binds to a distal enhancer of αA-crystallin (*C*yraa), DCR1, and regulates the expression of CYRAA (Yang et al., 2006). Further research shows that FGF signaling can upregulate c-Maf through c-Jun and Etv5/ERM, an AP-1 factor and an Ets factor, respectively, in a study of embryonic chick lenses (Xie et al., 2016). Similarly, in a study of chicks, BMP participated in LFC differentiation, with two BMP family members, namely, BMP4 and BMP7, being particularly active and endogenously expressed by lens cells (Boswell et al., 2008). BMP signaling acts as the intermediate regulator of FGF and crystallin genes, as indicated by abolishing BMP2/4/7 signaling by noggin impeding FGF-induced crystallin gene expression (Boswell et al., 2008). In addition, since cellular organelles, including the endoplasmic reticulum, Golgi apparatus, mitochondria, and nuclei, can interfere with lens transparency, organelle degradation is necessary. This degradation process is synchronized with the production of large amounts of crystallin protein in LFCs, which indicates well-established coordination of these two contradictory procedures. Apoptosis, autophagy, nucleophagy, and mitophagy pathways are all involved in organelle degradation (Wride, 2011; Costello et al., 2013). Wnt signaling also participates in LFC differentiation; in contrast to its role in the first stage, Wnt activation favors the differentiation of LFCs from LECs in this stage (Stump et al., 2003; Cain et al., 2008), indicating the positive role of Wnt in LEC transdifferentiation. A recent study of mice showed that a protein that targets mitochondria for elimination during erythrocyte formation, called BCL2-interacting protein 3-like (BNIP3L/NIX), is indispensable and participates in the formation of the lens organelle–free zone (Brennan et al., 2018). In summary, LFC differentiation is an integrated process that involves different normal cellular and molecular functions to maintain ongoing development.

Lens development is a stepwise process influenced by ubiquitous but stage-specific cellular and molecular mechanisms. Although tremendous progress has been made in deciphering lens morphogenesis, the evidence is fragmented and disconnected. The remaining gaps need to be filled before the completed blueprint of lens development can be precisely constructed. Based on the knowledge discovered to date, studies now focus on the interactions between molecular pathways at the microlevel and the corresponding coordination among different organelles at the cellular level. For instance, the negative regulation of PTEN on FGFR signaling during lens development was recently confirmed in mice (Padula et al., 2020). It has been discovered that hedgehog signaling (Taira et al., 2020), the ETV family (Garg et al., 2020), the SPREAD family (Wazin and Lovicu, 2020), and heat shock factors (Evans et al., 2007; Cui et al., 2020) are involved in lens development. Notably, these results were obtained from models of avian embryos, CHO cells, mice, and embryonic zebrafish. Although direct molecular regulation during lens development is important, the effects of the developmental microenvironment are also drawing increasing attention. In the following sections, common chemicals produced during multiple biological activities, ROS, and their related cellular and molecular influence on lens development are discussed.

## 3 ROS and the UPR

ROS, including superoxide anion (O_2_^−^), hydrogen peroxide (H_2_O_2_), and hydroxyl radical (HO•), are mainly generated during mitochondrial oxidative metabolism (Murphy, 2009) and other cellular processes in response to exogenous substances (Ray et al., 2012). They participate in ubiquitous activities ranging from physiological to pathological conditions. The development of multicellular organisms is one of the processes involving ROS. The balance between the proliferation and differentiation of stem cells determines the direction of development and can be dramatically influenced by ROS (Schippers et al., 2012). The cellular ROS concentration is in dynamic equilibrium, which requires the harmonious cooperation of both ROS production and their consumption by antioxidants, resulting in a delicate balance between the proper redox state and oxidative stress.

### 3.1 Main sources of cellular ROS

**FIGURE 1:**
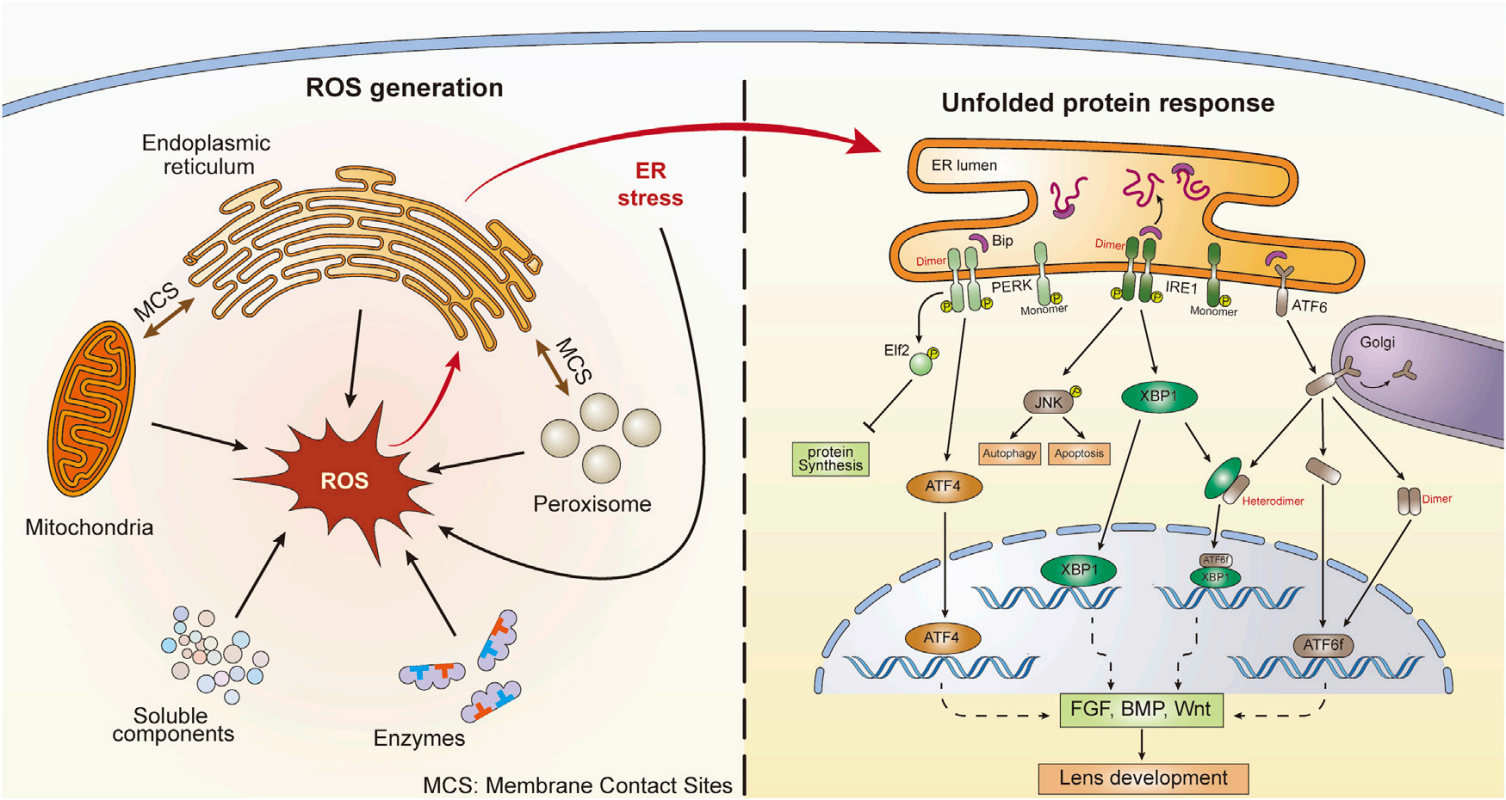
ROS generation and subsequent UPR activation in lens cells. There are five main ROS sources, including enzymes, soluble components, mitochondria, peroxisomes, and the endoplasmic reticulum (ER). ER stress and UPR can also generate ROS. There are membrane contact sites between ER and mitochondria as well as ER and peroxisomes. Three UPR signaling pathways are activated following ER stress, including ATF6, PERK, and IRE1. The dimerization and heterodimerization of key factors could lead to differential gene regulation. These UPR pathways might further influence lens development through action on the FGF, BMP, and Wnt pathways.

Cellular ROS can be generated in both the cytosol and organelles (Figure 1). Several soluble components, including flavins, thiols, catecholamines, and hydroquinones, can produce ROS during redox reactions (Freeman and Crapo, 1982). Additionally, during their catalytic activities, several ROS-producing cytosolic enzymes, such as xanthine oxidase, produce ROS (McCord et al., 1985). However, a large proportion of intracellular ROS originate from organelles, including mitochondria, peroxisomes, and the endoplasmic reticulum (ER). Mitochondria serve not only as the major energy-producing “factories” in aerobic cells but also as continuous ROS-generating “machines.” The transport of electrons along the respiratory chain is accompanied by the production of ROS (Sena and Chandel, 2012). In addition, several oxidoreductases located in mitochondrial membranes participate in ROS generation (Andreyev et al., 2005). Peroxisomes are organelles involved in the cellular metabolism of H_2_O_2_, a species that can be generated by urate oxidase and xanthine oxidase within peroxisomes (Angermuller et al., 1987; Fritz et al., 2007). Another important ROS-generating organelle is the ER, which is involved in multiple functions, such as protein synthesis and protein folding, calcium storage, and lipid metabolism (Koch, 1990; Groenendyk and Michalak, 2005). The electron transport chain in the smooth ER is sustained by xenobiotic metabolism and unsaturated fatty acid production. ROS can be produced during these activities (Di Meo et al., 2016). Additionally, the ER stabilizes proteins through the oxidative protein folding (OPF) reaction, for which molecular oxygen is the source of the oxidizing equivalents necessary to form intramolecular disulfide bonds. OPF is the most common posttranslational modification undertaken in the ER and is a major source of H_2_O_2_ therein (Malhotra and Kaufman, 2007; Qin et al., 2015). It has been estimated that oxidative protein folding within the ER accounts for up to 25% of cellular ROS production (Tu and Weissman, 2004). Moreover, membrane contact sites (MCSs) between organelles contribute to ROS generation. In mammals, more than 90% of peroxisomes and 20% of mitochondria contact the ER (Valm et al., 2017). Vesicle-associated membrane protein (VAMP)-associated proteins (VAPs) are one kind of MCS between the ER and other organelles (Murphy and Levine, 2016). Studies have shown that there are close connections between VAPs and the UPR signaling pathways (Kamemura and Chihara, 2019). It has been demonstrated that ROS production occurs at ER–mitochondria contact sites, which are conducted by calcium channels, namely, inositol 1,4,5-trisphosphate (IP3) receptors (Booth et al., 2016). These results indicate that MCSs between the ER and mitochondria or peroxisomes can contribute to the interplay between the UPR and OS. Additionally, a feedback loop exists between ROS and the UPR. Excessive production of ROS can lead to the accumulation of misfolded proteins in the ER and subsequent UPR activation. Interestingly, prolonged UPR activation promotes the accumulation of ROS (Haynes et al., 2004; Malhotra and Kaufman, 2007). The results showed that UPR-deficient cells under sustained ER stress are unable to accumulate ROS; however, cells with a functional UPR accumulate ROS, resulting in cell death (Haynes et al., 2004). The generation of ROS induced by the UPR in age-related cataracts is reported to be related to ERO1-Lα, ERO1-Lβ, and protein disulfide isomerase (Periyasamy and Shinohara, 2017). Metabolic processes also generate ROS, and the primary function of NADPH oxidases is to produce ROS. NADPH oxidases are composed of membrane-bound subunits and catalyze molecular oxygen reduction. When NADPH oxidases are activated, NADPH transfers its electrons to flavin adenine nucleotides. Subsequently, the electrons are passed to two heme groups bound to the N-terminal domain, which finally pass the two electrons to two molecular oxygen atoms on the opposite side of the membrane, forming superoxide anions (Bedard and Krause, 2007; Brieger et al., 2012).

### 3.2 Roles of ROS in development

In addition to directly producing biological effects, generated ROS can serve as signaling molecules that trigger active cellular responses. These responses are involved in the development of primitive embryos and subsequent organs (Coffman and Su, 2019; Lin and Wang, 2020). A recent study showed that early cleavage of *Xenopus* embryos is promoted by an increase in mitochondrial ROS levels (Han Y. et al., 2018), indicating the early intervention of ROS during embryogenesis. Considerable evidence has established that ROS participate in many processes during neurogenesis (Borquez et al., 2016; Oswald et al., 2018). Promoting the differentiation of stem cells and maintaining the stemness of neural precursors are two important roles of ROS (Wang et al., 2013; Chaudhari et al., 2014). In addition, ROS can shape the polarity of neurons. A study has shown that the ROS level is increased in polarizing cerebellar granule neurons but is decreased under depolarizing conditions (Olguin-Albuerne and Moran, 2015). Cytoskeletal modifications have also been shown to be linked to ROS, as reflected by the influence of cysteine and methionine residues on β-actin upon redox signaling (Lassing et al., 2007). ROS can also alter the ubiquitination of Yap, which is closely related to organ size, during neurogenesis (Ji et al., 2017). All of these aspects, the differentiation of progenitor cells, the establishment of polarity, the modification of the cytoskeleton, and the expansion of cell volume, play roles not only in neurogenesis but also in lens development (Donaldson et al., 2009; Muccioli et al., 2016; Cheng et al., 2017). Although the regulatory effect of ROS on lens development is currently unknown, experience from the redox-regulating factor glutathione provides some evidence about the influence of ROS on lens development. A recent study confirmed that impairing the biosynthesis of glutathione can disrupt eye development, including lens development (Thompson et al., 2021). In this way, we hypothesize that ROS also play roles in lens development, the details of which require future studies to determine.

### 3.3 The link between ROS and UPR

The homeostasis state of ROS in the ER is vital. Although OPF requires an oxidative environment to function effectively, excessive accumulation of ROS or excessive oxidative stress can disrupt homeostasis and lead to the accumulation of misfolded proteins in the ER, which causes ER stress (Tu and Weissman, 2004; Malhotra and Kaufman, 2007). In turn, under conditions of ER stress, the induced activity of the ERO1 (ER oxidoreductase 1)-PDI (protein disulfide isomerase) system accelerates the production of ROS by transferring electrons to molecular oxygen (O_2_) and producing H_2_O_2_ (Cao and Kaufman, 2014; Zeeshan et al., 2016). Perturbation results in UPR induction and initiates the activation of a series of subsequent signaling pathways that restore ER equilibrium (Figure 1) (Gardner et al., 2013).

#### 3.3.1 Initiation and modulation of the UPR

There are three UPR sensors distributed in the ER membrane, namely, activating transcription factor 6 (ATF6), protein kinase RNA-like endoplasmic reticulum kinase (PERK), and inositol-requiring protein 1 (IRE1) (Figure 1) (Zhang et al., 2019). ERO1 in the ER utilizes O_2_ to power the oxidization of PDI and generate H_2_O_2_, and this process forms a disulfide bond. However, when the concentration of H_2_O_2_ exceeds the threshold, redox homeostasis in the ER can be damaged. The OPF process is blocked and leads to the accumulation of unfolded proteins, which are seized by these three sensors to trigger different signaling pathways (Higa et al., 2014). Specifically, ATF6 is transported to the Golgi apparatus, where it is cleaved by two Golgi-resident proteases (Ye et al., 2000). Cleaved ATF6 (ATF6f) is further transported into the nucleus and acts as a transcription factor to promote the expression of activating transcription factor 4 (ATF4) and X-box-binding protein 1 (XBP1) (Yamamoto et al., 2007). PERK undergoes oligomerization and autophosphorylation when the UPR is initiated (McQuiston and Diehl, 2017) and then phosphorylates eukaryotic translation initiation factor 2α (eIF2α), which leads to selective expression of ATF4 (Rowlands et al., 1988). ATF4 is not only able to regulate adaptive genes that relieve ER stress but can also promote the expression of the CAAT/enhancer-binding protein homologous protein (CHOP), which participates in cell death induction (Rowlands et al., 1988; Han et al., 2013). After its autophosphorylation, IRE1α serves as an RNase and splices an intron of downstream *XBP1* mRNA, which is then translated into the potent transcription factor XBP1 and initiates the transcription of ER-quality-regulation genes (Maurel et al., 2014). Activation of the UPR signaling pathway can restore ER viability and is vital for cell growth and survival (Bertolotti et al., 2000). Normally, no UPR activity is triggered until ER homeostasis is challenged by ER stress-causing chemicals, such as excessive ROS. However, these UPR-mediating genes also participate in developmental behaviors in a number of species (Mitra and Ryoo, 2019), suggesting that the UPR can also function in normal physiological processes. Under conditions of ER stress, the UPR induces the expression of these genes to restore ER equilibrium.

The over- or prolonged activation of the UPR can have a harmful effect. Proper modulation of the UPR helps to neutralize detrimental effects. For example, in neurodegenerative diseases, research has proven that dysregulated UPR signaling is a major pathogenic mechanism. However, genetic modulation of the PERK axis in animal models can prevent neurodegeneration (Hughes and Mallucci, 2019). Interestingly, both inhibition and activation of the PERK pathway were found to be beneficial in neurodegenerative disease models (Shacham et al., 2021). In diabetes mellitus, overactivation of IRE1α triggered the apoptosis and degeneration of islet β-cells. Several IRE1α-inhibiting small molecule compounds spared β-cells from death (Ghosh et al., 2019). These results indicate that modulating the activation status of the UPR through genetic interference or compound addition can have advantages for disease treatment. Moreover, modulation of the UPR can help elucidate the exact interaction between ROS and the UPR. In a study using human osteosarcoma cells, ROS generation was downregulated after adding the PERK inhibitor GSK2606414 (Wang et al., 2019), indicating a ROS generation-promotion effect of the UPR in this model. In contrast, by using a curcumin derivative to produce ROS in cultured human amniotic mesenchymal stem cells, researchers found that the ROS level was further elevated after adding the PERK inhibitor GSK2656157 (Wang et al., 2021), indicating an adaptive response from UPR activation.

#### 3.3.2 Roles of ROS and the UPR in development

All three UPR branches are activated by the accumulation of misfolded proteins and/or the overload of unfolded proteins in the ER. The development of organisms is accompanied by an abundant synthesis of proteins, which requires the ER to operate under high loads. On the one hand, this load results in a dramatic increase in OPF, followed by the elevation of H_2_O_2_ levels. On the other hand, massive protein synthesis is more likely to lead to misfolded protein accumulation and the overload of unfolded proteins. Commonly, either situation can easily cause ER stress and UPR initiation signaling in developing cells. However, the roles of ROS and the UPR in development are far from clear. We currently know only the effect of core factors in the UPR signaling pathway.

There are two ATF6 species in vertebrate species, namely, ATF6α and ATF6β. Double knockout of *A*tf6α and *A*tf6β is lethal at the early embryonic stage, but single knockout of either type leads to more viable organisms (Yamamoto et al., 2007). Double-knockout mice present more severe malformations than mice with other types of UPR molecule knockouts, including *I*re1α, *X*bp1, *P*erk, or *A*tf4, indicating a broad role for the *A*tf6 gene in vertebrate development (Yoshida et al., 2000; Yamamoto et al., 2007). ATF6 participates in various developmental processes, including lens development (Firtina and Duncan, 2011; Clark et al., 2020), neurodevelopment (Saito et al., 2012; Naughton et al., 2015), mesoderm differentiation (Kroeger et al., 2018), osteogenesis and chondrogenesis (Maeda et al., 2015; Guo et al., 2016), adipogenesis and lipogenesis (Lowe et al., 2012), and the formation of the female reproductive system (Yang Y. et al., 2015; Xiong et al., 2015). Hyperactivation of ATF6 in human embryonic stem cells (hESCs) not only promotes the ER network but also facilitates mesodermal differentiation (Kroeger et al., 2018). In contrast, loss of ATF6 impedes the mesodermal fate of hESCs (Kroeger et al., 2018). In human eyes, mutation of *ATF6* leads to defects in the macular central fovea, which results in achromatopsia, an ocular disease characterized by a lack of cone photoreceptor function (Ansar et al., 2015; Kohl et al., 2015). Additionally, *ATF6* mutation can lead to cone-rod dystrophy. Patients suffering from this disease will gradually lose cone function followed by loss of rod function (Skorczyk-Werner et al., 2017). By using patient-specific iPSCs and retinal organoids, a recent study also showed that ATF6 is essential for the formation of human cone photoreceptors (Kroeger et al., 2021). All the evidence confirmed the important character of ATF6 during retinal development. Further studies confirmed that many different functions of ATF6, such as misfolded protein detection ability, were impaired upon *ATF6* mutation (Chiang et al., 2017; Tam et al., 2018). ATF6 also participates in lens development, which is discussed in the following section. Nevertheless, how the *ATF6* gene generally regulates the development of these ocular cells remains unclear.

Another UPR core organic macromolecule, PERK, also participates in the process of development. Loss-of-function mutation of *PERK* leads to a disease known as Walcott–Rallison syndrome, which is characterized by infantile-onset diabetes, exocrine pancreas dystrophy, and other problems in the bone, liver, and kidney (Durocher et al., 2006). Compared with that in adult tissues, the activation pattern of PERK/eIF2a is elevated in the brains of mouse embryos, indicating the participation of PERK during embryonic brain development (Zhang et al., 2007). PERK can affect miRNA networks associated with stemness and differentiation to determine the fate of myoblast differentiation. In one study of *P*erk knockdown in mice, myoblasts changed to stem-like cells with a marked transformation from a fusiform to a rounded morphology (Tan et al., 2021). A study in *P*erk-mutant β-islet cells showed that anterograde trafficking from the ER to the Golgi and retrotranslocation from the ER to the cytosol were impaired, as indicated by the reduced expression of ER chaperones (Gupta et al., 2010). In addition, a genome-wide association study (GWAS) revealed a set of human *PERK* variants in patients with progressive supranuclear palsy (PSP), which is characterized by abnormal tau deposits (Höglinger et al., 2011). However, the mechanism of *PERK*-mutant PSP is unclear because tau is not a secreted protein; thus, modification in the ER is unlikely. As the downstream factor of PERK, *A*tf4 deficiency can cause developmental defects, including pancreatic hypertrophy, in mice (Iida et al., 2007). *A*tf4-knockout mice also show defects in lens development. The details are discussed in the following section. However, the underlying mechanism by which PERK/ATF4 influences organ development remains unknown.

XBP1, generated by IRE1, is an important regulator during development. Mutation of *I*re1 or *X*bp1 in *Drosophila* is lethal at early larval stages. However, reintroduction of wild-type genes can reverse this effect (Huang et al., 2017). *X*bp1-knockout (*X*bp1^−/−^) mice die during embryogenesis because of liver dysfunction, indicating the requirement for XBP1 in liver development (Reimold et al., 2000). A study confirmed the requirement of spliced XBP1 (XBP1s) for the precise secretion of digestive enzymes in the *Drosophila* digestive tract (Huang et al., 2017). In fish, XBP1 is highly expressed in high-secretion cells, including those in the glands, notochord, and tail (Bennett et al., 2007; Ishikawa et al., 2017). XBP1s has been reported to be a differentiation factor required for hatching gland development (Bennett et al., 2007). In addition, XBP1s is the key factor for the growth and development of medaka fish because defects observed in XBP1-knockout or IRE1-knockout medaka are fully rescued by constitutive expression of XBP1s (Ishikawa et al., 2017). XBP1 also participates in the immune system response. *X*bp1^−/−^ mice fail to control infection with the B-cell-dependent polyoma virus because of the absence of plasma cells (Reimold et al., 2001). IRE1 shares some common features with XBP1 during development. *I*re1α-knockout mice present with defects in the liver and pancreas and salivary gland cells, similar to *X*bp1^−/−^mice (Iwawaki et al., 2010; Zhang et al., 2011). However, IRE1 also shows apparent differences with respect to XBP1. The development of the placenta in mice can be significantly influenced by IRE1α loss of function, while the developmental defects of the placenta in *X*bp1^−/−^ mice are moderate compared with those of *I*re1α^−/−^ mice (Iwawaki et al., 2009). This difference may result from the regulated IRE1-dependent decay (RIDD) of mRNA, which helps to maintain ER homeostasis by reducing the load of ER client proteins or inhibiting protein synthesis (Maurel et al., 2014). More importantly, IRE1 is active in early photoreceptors and is required for normal ER differentiation during the development of *Drosophila* photoreceptors (Xu et al., 2016). In addition, IRE1/XBP1 has been shown to be linked to retinitis pigmentosa (Chiang et al., 2015), indicating a connection between photoreceptors and IRE1 signaling. Since the generation of XBP1s can alleviate the protein-folding load in the ER, we hypothesize that the development-regulating effect of the IRE1–XBP1 axis might be part of a “production control” system. However, little is known about the underlying molecular mechanism.

In summary, the three UPR signaling pathways participate in developmental processes. Although some characteristics of different core factors in the UPR have been revealed, there is still a long way to go before the development-regulation blueprint of the UPR or the mutual relationship between the UPR and other canonical signaling pathways involved in development can be precisely outlined.

## 4 ROS and the UPR in lens development

As mentioned earlier, ROS play important roles in development, including the regulation of the proliferation and differentiation of stem cells (Holmstrom and Finkel, 2014). It has been shown that ROS are able to reduce the stemness of hESCs, promote their neural differentiation, and enhance the expression of PAX6 (Hu et al., 2018), an important factor that regulates lens development. ROS also directly influence the intracellular localization of PAX6. In corneal cells, PAX6 exhibits apparent nucleocytoplasmic shuttling following H_2_O_2_ treatment (Shukla and Mishra, 2018). Based on these results, we hypothesize that there might be a potential connection between ROS and lens development.

### 4.1 ROS and LECs

As an important type of lens cell, LECs may be closely related to ROS. Previous studies have shown that low levels of ROS can be generated following platelet-derived growth factor (PDGF) treatment of LECs (Chen et al., 2004; Wang and Lou, 2009). PDGF is a mitogenic factor that can promote the proliferation of LECs, in which ROS serve as signaling transducers to activate a series of downstream signaling pathways, including the MAPKs, ERK1/2, and JNK (Chen et al., 2004). It was confirmed that the upstream components of the PDGF receptor kinase, Src-family kinases, PI3K, RAC, RAS proteins, and arachidonic acid are required for ROS production and subsequent cell proliferation following PDGF treatment (Zhang et al., 2006; Chen et al., 2007). In addition, ROS are also involved in the cell-proliferation-promoting effect on other growth factors, including EGF and bFGF, in LECs (Ohguro et al., 1999). Importantly, these growth-factor-related proliferation-stimulating effects may be eradicated upon inhibition of ROS (both removal and blockade of ROS) (Zhang et al., 2008). Endogenous H_2_O_2_ is generated in LECs under basal conditions during lens development (Basu et al., 2014). We speculate that there is a basal developmental-regulatory role of ROS in LECs, which requires confirmation in future studies.

However, ROS can also contribute to cataract formation. We now understand that the homeostasis of the redox state in LECs is closely related to the transparency of the crystallin lens. The imbalance of free radical and antioxidant systems results in oxidative stress and subsequent cataracts (Wilhelm et al., 2007; Bai et al., 2013). Heme oxygenase-1 (*HO-1*) deficiency in LECs is an example of a stress-inducing condition. It has been shown that *H*o-1 mutant transgenic mice display cataracts 12 weeks after birth, and this phenotype can be extended into adulthood (Zhou et al., 2011). Our previous work confirmed that HO-1 can reduce ROS levels and increase antioxidant levels in LECs, which inhibits the apoptosis of LECs (Ma et al., 2016). As the major byproduct of HO degradation, carbon monoxide (CO) confers a protective effect similar to that of HO-1 on LECs following ROS treatment, as indicated in our recent study (Huang et al., 2018a). Pretreatment with CO can be used to target mitochondria in LECs and promote the return of normal redox homeostasis (Huang et al., 2018b). The detailed molecular mechanism of the interaction between ROS and HO-1 in LECs remains unknown. However, the major regulator of HO-1, nuclear factor erythroid 2-like 2 (NRF2), attracted our attention. NRF2 is a transcription factor that is critical for the regulation of redox reactions and antioxidants in mammalian cells (Kensler et al., 2007). Under physiological conditions, NRF2 is sequestered by the inhibitor protein Kelch-like ECH-associated protein 1 (KEAP1). When oxidative stress occurs, NRF2 is released from KEAP1, translocates to the nucleus, and initiates the expression of a series of genes, including HO-1 (Baird and Dinkova-Kostova, 2011). Our recent research showed that the expression level of NRF2 in LECs increased significantly following ROS treatment, and transfecting LECs with the *NRF2* gene enhanced the protective effect of NRF2 against oxidative damage (Ma et al., 2018). More importantly, NRF2 was found to interact with a UPR signaling pathway and the PERK pathway, by binding to the downstream factor ATF4. An increase in the NRF2-ATF4 complex level can be detected when the environmental H_2_O_2_ concentration increases, which facilitates NRF2-conferred protection (Ma et al., 2018). In summary, these results suggest a close relationship between ROS and LECs. Unfortunately, there is no direct research focusing on the potential effect of ROS on lens development, and future work is required to reveal the possible effect.

### 4.2 Cross-talk between components of the UPR and lens development

As an adaptive response to ROS, the UPR is established in the lens. The mechanism responsible for the activation of all three UPR pathways is highly conserved among vertebrates (Cvekl and Zhang, 2017), including mammals and fish (Ishikawa et al., 2011). These model animals are frequently used to study the influence of the UPR. There is considerable evidence showing UPR-related cataract formation (Torres-Bernal et al., 2014; Yang et al., 2015a; Yang et al., 2015b; Lyu et al., 2015) in these animals. However, in this situation, the UPR is highly activated, which induces apoptosis of both LECs and LFCs (Ikesugi et al., 2006). In contrast, moderate activation of the UPR was shown to influence normal lens development (Firtina et al., 2009). A study confirmed that all three UPR pathways are activated during mouse lens development but exhibit relatively different patterns (Firtina and Duncan, 2011).

**FIGURE 2:**
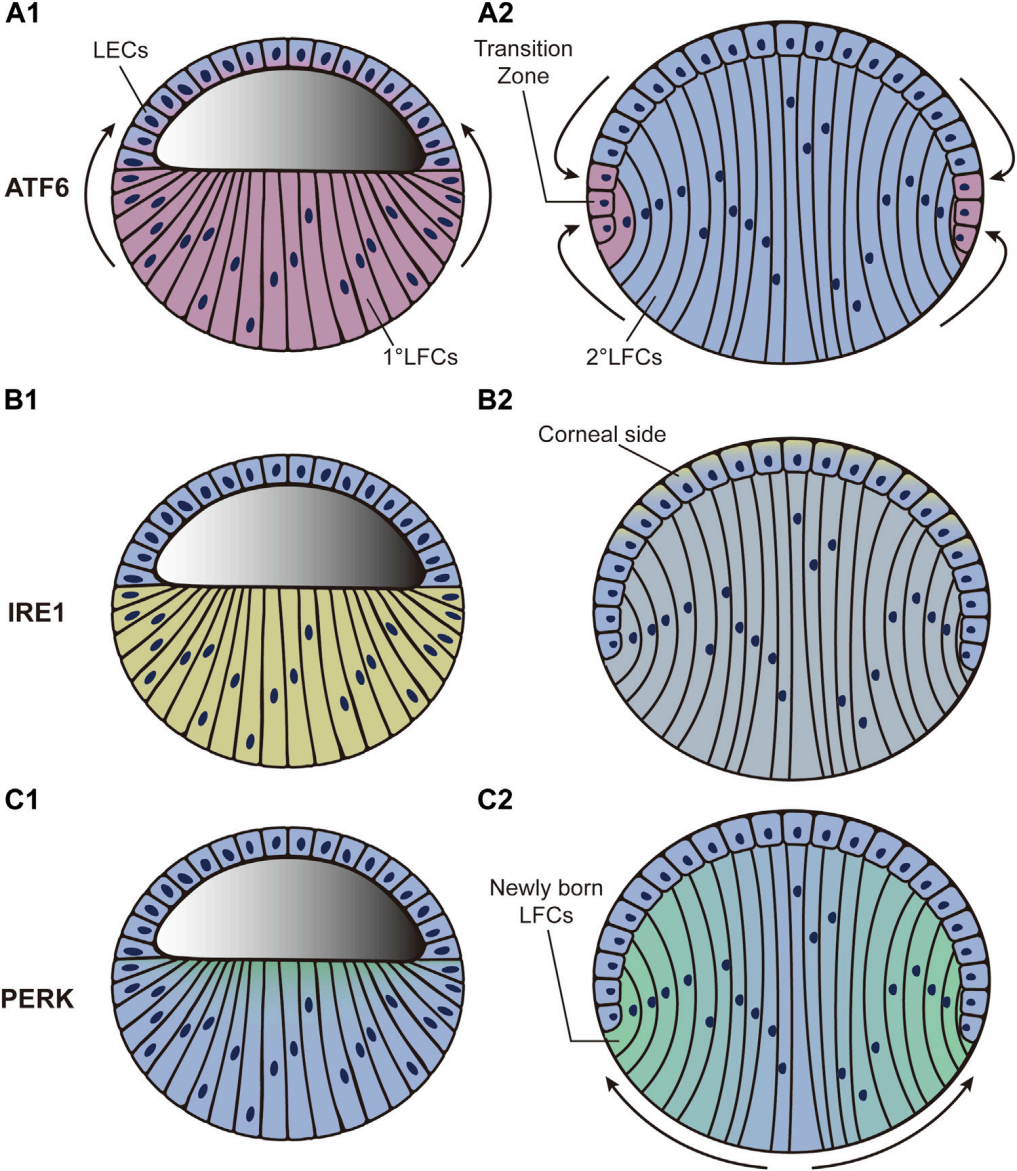
Dynamic distribution of three UPR pathways during the third phase of lens development. “Blue” refers to lens cells; “red,” “yellow,” and “green” in A, B, and C refer to ATF6, XBP1, and PERK expression, respectively. **(A**_**1**_ **–A**_**2**_ **)**: ATF6 expression level increases sharply in LFCs and gradually diffuses to LECs. By the end of lens development, the highest expression level of ATF6 is detected around the transition zone. **(B**_**1**_ **–B**_**2**_ **)**: XBP1 expression is initially increased in LFCs. It then decreases in LFCs but increases in LECs. By the end of development, the highest expression level of XBP1 is found in LECs adjacent to the corneal epithelium. **(C**_**1**_ **–C**_**2**_ **)**: PERK expression level first appears at the apical tips of all LFCs and then accumulates predominantly in newly born LFCs while declining to nothing in central LFCs as development proceeds. LECs: lens epithelial cells; LFCs: lens fiber cells.

ATF6, IRE1, and PERK are all expressed at low levels after the formation of lens vesicles (phase 2) in mice (Firtina and Duncan, 2011). As development continues, the ATF6 expression level increases sharply in LFCs and gradually diffuses in LECs. Through the end of lens development, the highest expression level of ATF6 is detected near the transition zone (Figures 2A_1_-A_2_) (Firtina and Duncan, 2011). To better investigate ATF6 activity during development, an ATF6-eGFP reporter was recently created for use in zebrafish (Clark et al., 2020). After injecting the reporter plasmid into zebrafish embryos, the highest eGFP expression was observed in the lens and skeletal muscle. The expression in skeletal muscle decreased as development continued, while the expression in lens remained relatively consistent (Clark et al., 2020). This exciting result indicates the full participation of ATF6 during lens development. However, current studies only provide us with correlative evidence. To further speculate on the underlying mechanism, we referred to the molecular function of ATF6. A previous study has shown that ATF6 is closely linked to tolerance to chronic stress (Wu et al., 2007). The subunit activity of ATF6 also plays important roles in maintaining tissue homeostasis (Hillary and FitzGerald, 2018). During the late stage of lens development, LFCs undertake extensive crystallin protein synthesis. Simultaneously, the elongation of LFCs requires multiple microtubules to promote dramatic deformation (Audette et al., 2017). The differentiation from LECs to LFCs at the transition zone is accompanied by a series of changes in gene expression patterns. As massive protein synthesis, microtubule movements, and changes in gene expression patterns might disturb homeostasis within lens cells, we hypothesize that the expression pattern of ATF6 during lens development could guarantee the relative stabilization of the internal microenvironment.

The expression level of XBP1, the downstream factor of IRE1, is also increased in LFCs during development (Firtina and Duncan, 2011). It then decreases in LFCs but increases in LECs during the middle stage (phase 3). By the end of development, the highest expression level of XBP1 is found in LECs adjacent to the corneal epithelium (Figures 2B_1_-B_2_) (Firtina and Duncan, 2011). Unfortunately, how the IRE1 branch actually influences lens development is far from clear. We presume that the role of IRE1 is linked to protective effects. Deletion of *X*bp1 in epithelial cells dramatically inhibits cell proliferation and differentiation as well as characteristic protein synthesis in mice (Hasegawa et al., 2015), indicating the necessity of XBP1 in epithelial growth. In *Drosophila*, a study confirmed the physiological stress resolution characteristics of the IRE1–XBP1 axis during normal development (Huang et al., 2017). More specifically, IRE1–XBP1 was strongly activated in neuropil and peripheral glia in *Drosophila* and the counterparts of oligodendrocytes and Schwann cells in mammals, providing protective and homeostasis-maintaining effects to nervous systems (Sone et al., 2013). Additionally, activation of IRE1 was related to the activation of the proapoptotic JNK pathway, which can lead to apoptosis (Urano et al., 2000). As LECs are located at the frontal surface of the lens and directly face the aqueous humor and corneal epithelium, we hypothesize that the accumulation of IRE1 in LECs might protect cells against apoptosis and help maintain homeostasis in lens development.

The participation of PERK starts in the third phase of lens development. The PERK expression level first appears at the apical tips of all LFCs and then becomes predominant in new LFCs but is completely abolished in the central LFCs as development proceeds (Figures 2C_1_-C_2_) (Firtina and Duncan, 2011). As a downstream factor in the PERK pathway, ATF4 has also been shown to play an important role in lens development. *A*tf4-knockout mice display impaired secondary LFC formation, while their primary LFCs and normal retinal development seem to be unaffected (Tanaka et al., 1998). Histological analysis showed that LFCs undergo apoptosis as development proceeds. The failure of secondary LFC formation ultimately results in microphthalmia (Tanaka et al., 1998). To further determine whether *A*tf4 gene expression in the lens is essential for LFC differentiation, the *A*tf4 gene was transferred downstream of the αA-crystallin promoter in *A*tf4^−/−^ mice. The microphthalmia defect was fully ameliorated in transgenic mice (Tanaka et al., 1998). In addition, a study based on transcriptomic analysis revealed the potential controlling role of eIF2 during lens development (Zhao Y. et al., 2018). These results are in agreement with the characteristics of the PERK activation pattern during lens development. In contrast to ATF6 and IRE1, which both induce a transcriptional response to relieve stress conditions, PERK mainly causes a rapid reduction in global rates of protein translation (Hughes and Mallucci, 2019). The conversion of LECs into LFCs is coupled with the elongation of the cell body together with the synthesis of crystallin protein. We hypothesize that PERK-ATF4 activation might help to stop irrelevant protein synthesis and minimize energy consumption.

Currently, there are no direct studies showing the influence of the UPR on signaling pathways that participate in lens development. However, the UPR is connected to these signaling pathways in other types of cells. As mentioned in the second section, FGF, BMP, and Wnt serve as core molecular regulators in three lens development stages. A study found that UPR activation is an inducer of FGF1 and FGF2 expressions in melanoma cells and is positively correlated with the activity of the ATF6 and PERK signaling pathways (Eigner et al., 2017). During angiogenesis, PERK kinase and its downstream factor ATF4 are drivers of FGF2 expression (Wang et al., 2012; Philippe et al., 2016). Additionally, moderate UPR activation can lead to the expression of FGF21 and its receptor FGFR1 in cardiomyocytes (Liang et al., 2017). The IRE1α–XBP1 pathway directly activates the transcriptional expression of *F*gf21 (Jiang et al., 2014). However, in contrast to typical ROS-mediated UPR activation, hypoxia-induced UPR activation reduces FGF signaling in cardiac progenitor cells (Shi et al., 2016), indicating that hypoxia-induced UPR plays a different role than that of the ROS-mediated UPR. For BMP signals, an interconnected regulatory loop is established with XBP1 and BMP4 in *Xenopus* that synergistically participates in mesodermal and neural tissue development (Cao et al., 2006). However, studies are now focused more on BMP signaling as an upstream factor that initiates activation of the UPR during osteogenesis, not the downstream effector (Li et al., 2014; Tanaka et al., 2014). We speculate that BMP signals might also play a regulatory role through UPR signaling pathways during lens development, for which further studies are needed for verification. Wnt has also been linked to the UPR. In osteocytes, genome-wide RNA sequencing indicates a regulatory effect of upstream XBP1 on Wnt signaling (Zhou et al., 2018), and UPR activation may result in active Wnt signaling (Chan et al., 2017). In stem cell differentiation studies, UPR activation drives endodermal cell fate via the β-catenin signaling pathway (Xu et al., 2014). ATF4 and ATF6 were found to be linked with the β-catenin pathway during osteogenesis and adipogenesis, respectively (Nakamura et al., 2013; Yu et al., 2013). In some cancer cells, IRE1α–XBP1 plays a positive regulatory role in the Wnt signaling pathway (Hua et al., 2013; Rodvold et al., 2017). Based on the relevant evidence, we hypothesize that there might be a positive correlation between the UPR and Wnt signaling in lens development.

Taken together, the data indicate that the three branches of the UPR may participate in lens development and display potential interconnections with the signaling pathways involved. However, current evidence is still fragmented. Only several core molecules of the UPR have been demonstrated to participate in lens development. Since some factors, such as ATF4, are multifunctional, studies based on individual factors within the UPR cannot illustrate the precise mechanism by which the UPR influences lens development. Additionally, lens development is a continuous process that occurs in fetuses in the uterus. It is relatively difficult to acquire direct and timely morphological and molecular evidence to better understand developmental processes. Because of ethical problems, current knowledge is largely based on model animals that are profoundly different from humans. Although capturing the blueprint of the UPR molecular regulation pattern and finding direct evidence of human lens development are of great importance, selecting an appropriate model seems to have become a priority.

## 5 Future directions

Great progress has been made in recent regenerative medical studies. hESC- and human-induced pluripotent stem cell (hiPSC)-based 3D tissue generation has shed light on developmental biology. These tools not only provide new ways to elaborately study both morphological and molecular details during human organ formation but also serve as ideal models to study diseases and screen drugs (Rossi et al., 2018). Currently, using hESCs or hiPSCs, stem cell technology is applied to generate organ-like structures (organoids), including the optic cup, cerebrum, cerebellum, intestine, colon, liver, lung, and kidney (Eiraku et al., 2008; Spence et al., 2011; Nakano et al., 2012; Fordham et al., 2013; Takebe et al., 2013; Takasato et al., 2014; Muguruma et al., 2015; Dye et al., 2016). Cultivated tissues show organizational structures, cell composition, and functional characteristics similar to those of the original organs. Based on the 3D cultivation system, in our previous studies, we successfully analyzed the influence of microenvironmental changes during retinal development (Gao et al., 2016) and recently isolated a group of promising therapeutic cell sources to treat retinal degenerative diseases (Zou et al., 2019).

**FIGURE 3:**
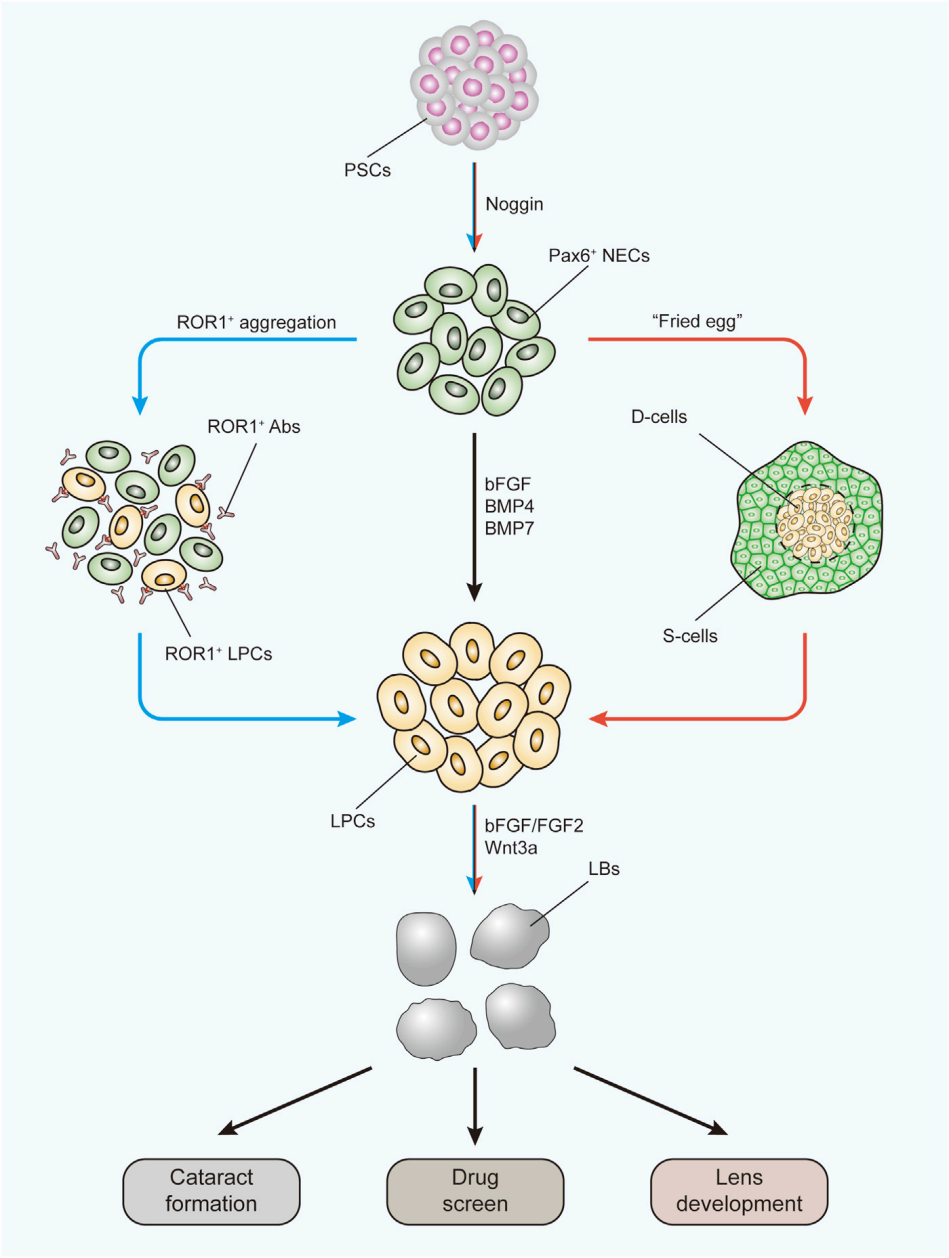
Three-stage protocol to form LBs from human PSCs using two improved methods. The black arrow indicates the main route of the three-stage protocol. The blue arrow indicates the ROR1^+^ LPC aggregation methods. The red arrow indicates the “fried egg” method. LBs generated under these protocols can be used to study the mechanism of cataract formation, to apply drug screens, and to investigate lens development. PSCs, pluripotent stem cells; NECs, neuroectodermal cells; Abs, antibodies; LPCs, lens progenitor cells; D-cells, differentiating cells; S-cells, supporting cells; LBs, lentoid bodies.

Due to the special construction of the lens, the ideal 3D cultivation method for generating intact and functional human lenses has not yet been developed. However, efforts are being made to form lens-like (lentoid) bodies or microlenses from hESCs and hiPSCs (Figure 3). Yang et al. (2010)first reported a three-stage system to differentiate hESCs into lens progenitor cells that subsequently formed small lentoid bodies (approximately 1,000 bodies per 30-mm well). Considering this result, Murphy et al. (2018)successfully generated microlenses with light-focusing ability, the characteristic function of the lens, from both hESCs and hiPSCs by aggregating ROR1^+^ LECs from induced lens progenitor cells. Later, the antibody isolation method was simplified through research and development of the cell strainer method (Dewi et al., 2020). The cells generated with this method showed RNA expression profiles similar to those of ROR1^+^ LECs and could also generate lentoid bodies with light-focusing ability (Dewi et al., 2020). A “fried egg” method was created by Fu et al., (2017a) in 2017. In this protocol, hiPSCs were induced into lens progenitor cells and lentoid bodies. This method significantly increased the induction efficiency and volume of the lentoid body (diameter approximately 3 mm). Further transcriptional analysis confirmed that the expression profiles of both the hESC- and hiPSC-derived lentoid bodies generated with the “fried egg” protocol were comparable (Ali et al., 2019) and mimicked the early stage of lens development (Ali et al., 2020). Nevertheless, the “fried egg” method may be regarded as an upgrade of the three-stage protocol, as the induction time window and induction supplement are relatively identical. In contrast, Nanog-negative peripheral cells were isolated at the end of the first step in the “fried egg” method. Both the ROR1^+^ cell aggregation and “fried egg” methods are enriched in LEC progenitor cells at second-stage induction, which contributes to lens fate establishment from ESCs and iPSCs.

These models were proven to be excellent for exploring cataract formation mechanisms (Murphy et al., 2018; Qin et al., 2019) and studying autophagy during lens development (Fu et al., 2017b). More importantly, as they are more disposable and because the results are easier to monitor than *in vivo* models, these models are ideal by their nature for studying the influence of specific signaling pathways during human lens development (Figure 3). By using the “three-stage” protocol, a study confirmed the lens differentiation-promoting effect of the noncanonical Wnt pathway (Han C. et al., 2018). As the UPR is also involved in signaling pathway activation, these models can be similarly used to study the UPR influence. The pathological effects of the UPR have already been discovered using kidney, lung, and intestinal organoids (Dvela-Levitt et al., 2019; Bampi et al., 2020).

Additionally, progress in other technical areas also helps the study of lens development. Emerging high-throughput methods, including microarrays, RNA-sequencing, and tandem mass spectrometry, provide us with better approaches to understand the gene regulatory network and generate a global protein expression profile during lens development (Anand and Lachke, 2017; Aryal et al., 2020). Advances in single-cell transcriptomics further expand research tools. Using this technology, Dylan et al. discovered the temporospatial expression patterns of three crystallin proteins and examined the expression dynamics related to cytoskeletal, RNA-binding, membrane-associated, and transcription factor genes in the zebrafish lens (Farnsworth et al., 2021). With this technical progress, the technology of 3D tissue generation can be better used for future research on lens development.

In summary, it is foreseeable that lentoids derived from hESCs or hiPSCs will be able to provide a new and relatively direct understanding of the UPR in lens development and may become a momentous tool for development biology research.

## 6 Conclusion

Lens development and UPR signaling pathways have been elaborately investigated in recent decades. The former is usually considered a normal physiological process, while the latter is often studied under pathological conditions. However, growing evidence has demonstrated that some core factors of the UPR are also important during developmental processes, although errors in the UPR function can turn “physiological” into “pathological.” To date, there is still a large gap in understanding the effects of the UPR during lens development. Current studies only provide correlative evidence based on individual factors in the UPR. The detailed interrelations as well as the specific influence on each developmental stage will require extensive efforts to develop the precise blueprint. Indeed, the number of developing lens cells is small, in addition to unknown processes during lens development, which both become critical challenges faced by future research. As the novel 3D cultivation method continues to be developed, there is reason to believe that dramatic progress can be made with this technological advance. More importantly, the orchestrating but concise construction, simple cellular constitution, and easy monitoring methods make the lens a perfect model for deciphering developmental codes. Thus, determining the ROS and UPR mechanisms in lens development is of great value not only within the ophthalmological field but also for developmental research.

## Glossary

aPPR: anterior pre-placodal region
ATF4: activating transcription factor 4
ATF6: activating transcription factor 6
BMP: bone morphogenetic protein
BNIP3L: BCL2-interacting protein 3-like
CHOP: CAAT/enhancer-binding protein homologous protein
CO: carbon monoxide
CRYAA: αA-crystallin
DCR: distal control region
eIF2α: eukaryotic translation initiation factor 2α
ER: endoplasmic reticulum
ERK1/2: extracellular signal-regulated kinases 1 and 2
ERO1: endoplasmic reticulum oxidoreductin 1
F-actin: filamentous actin
FGF: fibroblast growth factor
FSR2: fibroblast growth factor receptor substrate 2
GRB2: growth factor receptor-bound protein-2
GWAS: genome-wide association study
hESC: human embryonic stem cell
hiPSC: human induced pluripotent stem cell
HO-1: heme oxygenase-1
IRE1: inositol-requiring protein 1
JNK: c-Jun N-terminal kinase
Keap1: Kelch-like ECH-associated protein 1
LEC: lens epithelial cell
LFC: lens fiber cell
MAPK: mitogen-activated protein kinase
MCS: membrane contact site
NADPH: nicotinamide adenine dinucleotide phosphate
NRF2: nuclear factor erythroid 2-like 2
OPF: oxidative protein folding
OTX2: orthodenticle homeobox 2
PAR3: partitioning defective protein 3
PAX6: paired box protein 6
PDGF: platelet-derived growth factor
PDI: protein disulfide isomerase
PERK: protein kinase RNA-like endoplasmic reticulum kinase
PI3K: phosphoinositide 3-kinase
PSP: progressive supranuclear palsy
PTEN: Phosphatase and tensin homolog
RhoA: Ras homolog family member A
RIDD: regulated IRE1-dependent decay
ROR1: receptor tyrosine kinase-like orphan receptor 1
ROS: reactive oxygen species
RTK: receptor tyrosine kinase
SHP2: Src homology 2 domain-containing protein tyrosine phosphatase 2
UPR: unfolded protein response
XBP1: X-box-binding protein 1
Yap: Yes-associated protein.
